# Structure and Assembly Properties of the N-Terminal Domain of the Prion Ure2p in Isolation and in Its Natural Context

**DOI:** 10.1371/journal.pone.0009760

**Published:** 2010-03-22

**Authors:** Luc Bousset, Jonathan Bonnefoy, Yannick Sourigues, Frank Wien, Ronald Melki

**Affiliations:** 1 Laboratoire d'Enzymologie et Biochimie Structurales, CNRS, Gif-sur-Yvette, France; 2 Synchrotron Soleil, Gif-sur-Yvette, France; Dalhousie University, Canada

## Abstract

**Background:**

The aggregation of the baker's yeast prion Ure2p is at the origin of the [*URE3*] trait. The Q- and N-rich N-terminal part of the protein is believed to drive Ure2p assembly into fibrils of amyloid nature and the fibrillar forms of full-length Ure2p and its N-terminal part generated *in vitro* have been shown to induce [*URE3*] occurrence when introduced into yeast cells. This has led to the view that the fibrillar form of the N-terminal part of the protein is sufficient for the recruitment of constitutive Ure2p and that it imprints its amyloid structure to full-length Ure2p.

**Results:**

Here we generate a set of Ure2p N-terminal fragments, document their assembly and structural properties and compare them to that of full-length Ure2p. We identify the minimal region critical for the assembly of Ure2p N-terminal part into amyloids and show that such fibrils are unable to seed the assembly of full length Ure2p unlike fibrils made of intact Ure2p.

**Conclusion:**

Our results clearly indicate that fibrillar Ure2p shares no structural similarities with the amyloid fibrils made of Ure2p N-terminal part. Our results further suggest that the induction of [*URE3*] by fibrils made of full-length Ure2p is likely the consequence of fibrils growth by depletion of cytosolic Ure2p while it is the consequence of *de novo* formation of prion particles following, for example, titration within the cells of a specific set of molecular chaperones when fibrils made of Ure2p N-terminal domain are introduced within the cytoplasm.

## Introduction

Prions are infectious proteins [1]. In the baker's yeast *Saccharomyces cerevisiae* they are at the origin of the occurrence, maintenance, transmission in a non-mendelian manner and propagation of 3 traits, the [*URE3*], [*PSI^+^*] and [*PIN^+^*] phenotypes [2]–[4]. As prion emergence is neither accompanied by changes in the chemical nature of the infectious protein nor by its cleavage/degradation, it is widely believed that prion “infectivity” is the consequence of structural rearrangement of the prions leading to sustained function alteration and aggregation [5]. Primary structure analysis reveals that the three prions identified so far in yeast possess domains with unusual amino acid composition. Indeed these domains that can be N- or C-terminal are unusually rich in Q and N and to a lesser extent G, S and T residues [6]. While these domains are poorly structured in the constitutive form of prions, they are subject to conformational rearrangements in the form of the protein associated to the prion traits [7]–[9]. A number of genetic screens highlighted the importance of these domains for prion traits occurrence and propagation. Deletion studies have also revealed that the functional domains of the proteins affect prion conversion and allowed delineating minimal prion domains which overexpression, in a context where full-length prions are expressed, induces very significantly and efficiently prion phenotypes [10]–[12].

The prion domain of Ure2p is N-terminal and spans residues 1–93 [13]. A shorter fragment spanning residues 1–65 induces [*URE3*] [14]. *In vitro*, under physiological conditions, full-length Ure2p assembles into helical twisted fibrils that are ∼20 nm wide [13], [15] that lack amyloid signature [7], [16]–[20] while the prion domain in isolation assembles into ∼5 nm wide amyloid fibrils [20]–[21]. Comparison of the assembly kinetics of full-length Ure2p and Ure2p 1–93 reveals that the N-terminal domain assembles into protein fibrils faster and more readily than the full-length protein [6]. Both fibrils induce [*URE3*] when reintroduced in yeast cells [22] suggesting that both kinds of fibrils seed the assembly of constitutively expressed Ure2p.

To further narrow the length of the prion domain to the minimal size necessary and sufficient for assembly into fibrils we generated a set of Ure2p N-terminal fragments and documented their assembly properties and the structural characteristics of the assemblies. We show that the N-terminal 42 amino acid residues of Ure2p do not assemble into fibrils that bind thioflavin T while the peptides 1–79 and 1–93 do. This suggests that the minimal region critical for assembly into fibrils spans amino acid residues 43–79. We show that this polypeptide which N content is extremely high (57% of amino acid residues) assembles indeed into fibrils.

To determine whether the induction of [*URE3*] upon reintroduction of fibrils made of Ure2p N-terminal domain is the consequence of Ure2p 1–93 seeded assembly of endogenous Ure2p or due to *de novo* aggregation of endogenous Ure2p, we documented the elongation capacities of preformed Ure2p 42–79, 1–79 and 1–93 fibrils in the presence of soluble full-length Ure2p. We show that fibrils made of the N-terminal domain of Ure2p are devoid of seeding capacities unlike those made of the full-length protein. Our data suggest that the induction of [*URE3*] by fibrils made of Ure2p N-terminal domain is the consequence of *de novo* formation of prion particles following, for example, titration within the cells of a specific set of molecular chaperones by the fibrils. The inability of Ure2p 42–79, 1–79 and 1–93 fibrils to seed the assembly of soluble full-length Ure2p further suggest that fibrils made of authentic Ure2p share neither a scaffold nor structural similarities with Ure2p 42–79, 1–79 and 1–93 amyloids.

## Results

### Structure and Assembly of Ure2p prion domain fragments

**Figure 1:**
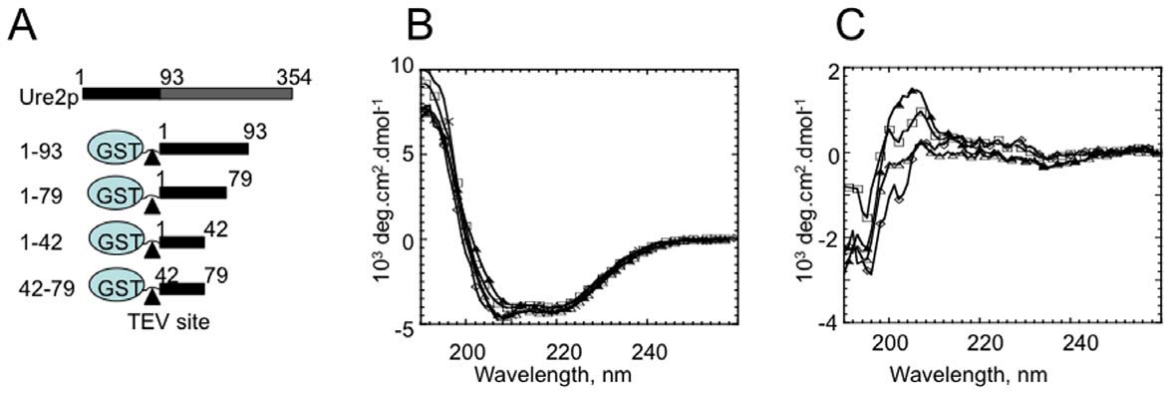
Characterization of recombinant Ure2p and its N-terminal fragments. **A**, Schematic representation of full-length Ure2p (the flexible N-terminal domain is coloured black while the compactly folded C-terminal domain is coloured grey) and the different fragments of its N-terminal domain expressed fused to GST. The numbers refer to amino acid residues. GST is shown as a circle. The TEV cleavage site engineered between GST and the N-terminal fragments of Ure2p is indicated by an arrow-head. **B**, CD spectra of GST alone (×), GST-Ure2p 1–93 (⋄), GST-Ure2p 1–79 (▵), GST-Ure2p 42–79 (□) and GST-Ure2p 1–42 (▴). **C**, CD difference difference spectrum between GST and GST-Ure2p 1–93 (⋄), GST-Ure2p 1–79 (▵), GST-Ure2p 42–79 (□) and GST-Ure2p 1–42 (▴).

**Figure 2:**
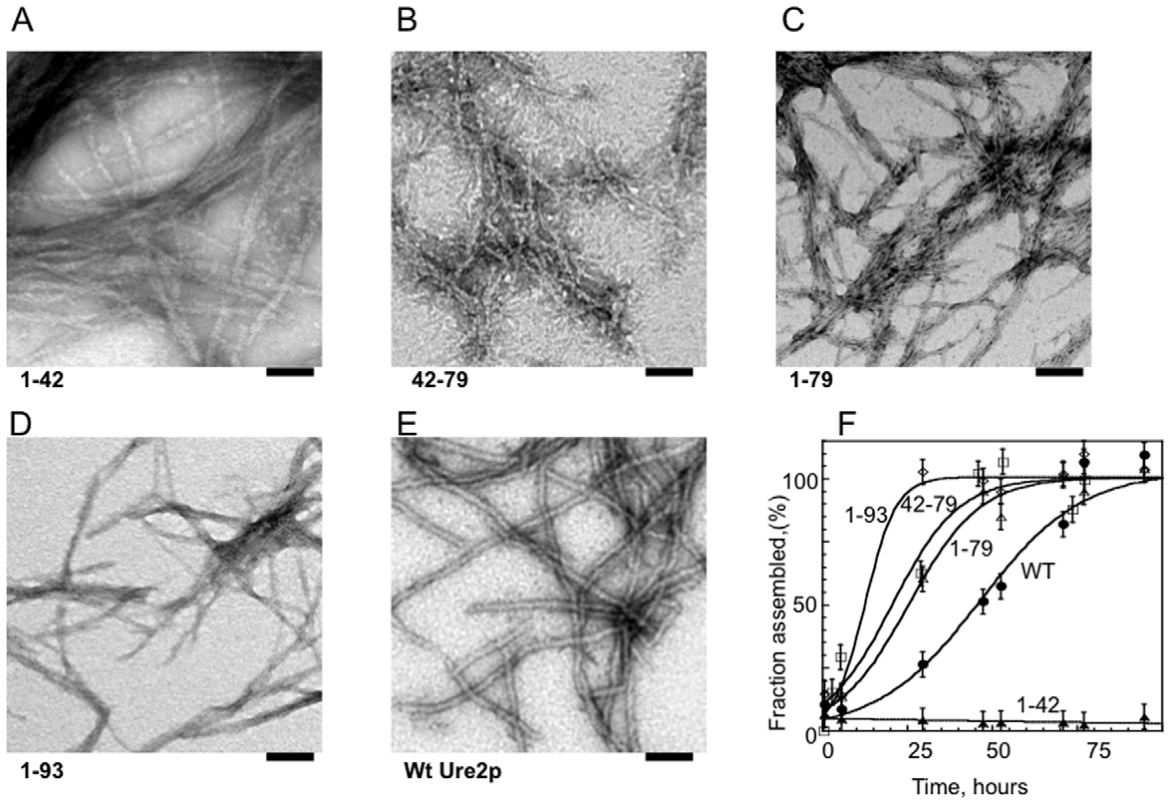
Ultra-structural and kinetic characterization of Ure2p 1–42, Ure2p 42–79, Ure2p 1–79, Ure2p 1–93 and full-length Ure2p assemblies. Negatively stained electron micrographs of Ure2p 1–42, Ure2p 42–79, Ure2p 1–79, Ure2p 1–93 and full-length Ure2p assemblies generated upon incubation of the different polypeptides (40 µM) for 80 h at 12°C in buffer A are shown in **A–E**, respectively. The scale bar in **A–E** represents 100 nm. **F**, assembly kinetics at 12°C of full-length Ure2p (•), Ure2p 1–42 (▴), Ure2p 42–79 (□), Ure2p 1–79 (▵) and Ure2p 1–93 (⋄), 40 µM, at 12°C in buffer A followed by Thoflavin T binding. The error bars indicate the standard deviation in three independent measurements.

We previously reported that the prion domain of Ure2p is flexible [13], [23]. GST-Ure2p 1–42, 42–79, 1–79, 1–93 (Ure2p1–42, 43–79, 1–79 and 1–93 correspond to Ure2p N-terminal fragments spanning amino acid residues 1 to 42, 43 to 79, 1 to 79 and 1–93, respectively) and glutathione S-transferase (GST) alone exhibit similar far UV circular dichroism (CD) spectra (Figure 1B). The CD difference spectra of GST-Ure2p 1–42, 42–79, 1–79, 1–93 and GST shows that the absence of Ure2p N-terminal fragments results in a decrease of the ellipticity signal between 200 and 190 nm, indicating that Ure2p 1–42, 42–79, 1–79, 1–93 remain in a random coil conformation within the fusion polypeptides (Figure 1C). Pure, free Ure2p 1–42, 42–79, 1–79, 1–93 assemble spontaneously under physiological pH and ionic strength into long unbranched fibrils (Figure 2A–D, respectively) in a manner similar to full-length Ure2p (Figure 2E). Interestingly however, while full-length Ure2p, Ure2p 42–79, 1–79 and 1–93 bind thioflavin T (ThT) and exhibit a typical sigmoid assembly curve, Ure2p 1–42 does not bind ThT (Figure 2F). Comparison of the assembly kinetics of free Ure2p 42–79, 1–79 and 1–93 and full-length Ure2p show that the free N-terminal fragments of Ure2p assemble much faster that the authentic protein (figure 2F), while GST-Ure2p 42–79, 1–79 and 1–93 assemble slower than full-length Ure2p (not shown). The electron micrographs of negatively stained samples of free Ure2p 1–42, 42–79, 1–79, 1–93 and full-length Ure2p incubated for 90 h at 6°C reveal that all polypeptides, including Ure2p 1–42, assemble into fibrils that are 5–10 nm in diameter while full-length Ure2p fibrils and fibrils obtained upon incubation of GST-Ure2p 1–42, 42–79, 1–79 and 1–93 for 150 hours are thicker (25 nm wide, Figure S1). Furthermore, while fibrillar full-length Ure2p, GST-Ure2p 1–42, 42–79, 1–79 and 1–93 have curly appearance, Ure2p 1–42, 42–79, 1–79 and 1–93 are stiff. The finding that Ure2p 1–42 assembles into fibrils indistinguishable from fibrillar Ure2p 42–79, 1–79, 1–93 is surprising as assembly is not accompanied by ThT binding.

### Secondary structure content of fibrillar Ure2p 1**–**42, 42**–**79, 1**–**79 and 1**–**93

**Figure 3:**
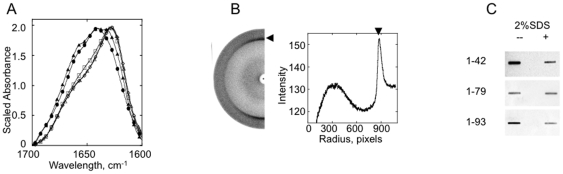
Structural characterization of fibrillar Ure2p 1–42, Ure2p 42–79, Ure2p 1–79, Ure2p 1–93 by FTIR spectroscopy, X-ray fiber diffraction and filter trapping. **A**, FTIR spectra of fibrillar Ure2p 1–42 (▴), Ure2p 42–79 (□), Ure2p 1–79 (▵), Ure2p 1–93 (⋄) and full-length Ure2p (•). **B**, X-ray diffraction pattern of fibrillar Ure2p1-93 (left side of the panel). The pattern shows a sharp refexion at 4.7 Å (black arrow head) that exhibits an increased intensity along the meridian and that characterizes amyloid fibrils. The X-Ray diffraction pattern was analysed using FibreFix version 1.3.1 software. Circular average was achieved over 180 degrees, the angular average is shown as a function of the radius in the right side of the panel. **C**, the resistance of fibrillar Ure2p 1–42, 1–79 and 1–93 to SDS (2%) was assayed by filtration through cellulose acetate membranes (0.20 µm pore size, Millipore Corp., Bedford, MA, USA). The filter was stained with amido black.

Previous results have shown that full-length Ure2p fibrils assembled under physiologically relevant conditions exhibit Fourier Transform Infrared Spectroscopy (FTIR) spectra typical of proteins with high helical content [16]–[17]. Fibrillar Ure2p 42–79, 1–79 and 1–93 exhibit FTIR spectra in deuterium oxide (D_2_O) typical of β-sheet rich amyloids and dominated by a strong absorbance at 1625 cm^−1^ (Figure 3A). Fourier deconvolution of fibrillar Ure2p 1–79, 42–79 and 1–93 indicates a β-sheet content of over 38% (Figure S2 A–D, Table 1). The latter are organized into a cross-β core as revealed by the fibre X-ray diffraction pattern that shows a sharp equatorial reflexion at 4.7 Å and a fuzzy reflexion at 10 Å suggesting partial disorder in the β-sheet packing within the fibrils (Figure 3B).

**Table 1:** Secondary structure content of fibrillar full-length Ure2p, Ure2p 1–94, 1–79, 1–42 and 42–79 estimated from the deconvolution of the FTIR spectroscopic measurements presented in Figure S2.

	α-helix [%]	β-sheet [%]	Other [%]
Ure2p 1–94	16	37	47
Ure2p 1–79	21	38	41
Ure2p 1–42	16	15	69
Ure2p 42–79	21	25	54
Ure2p WT	35	16	49

Interestingly the FTIR spectra of polymerized Ure2p 1–42 lies between the helical spectrum of full-length Ure2p and that of the amyloid forming peptides Ure2p 42–79, 1–79 and 1–93 with an α-helical content of 16% and a β-sheet content 15% (Figure S2 E, Table 1). The amyloid nature of fibrillar Ure2p 1–42, 42–79, 1–79 and 1–93 was further confirmed by the finding that they resist SDS treatment (figure 3C).

### Secondary structure changes during the assembly of Ure2p 1**–**79

**Figure 4:**
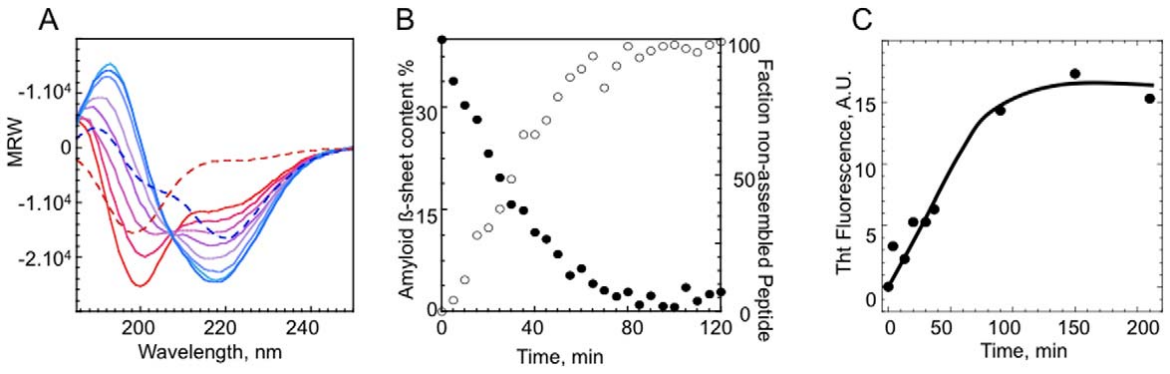
Secondary structure changes accompanying the assembly of Ure2p 1–79. **A**, Time course of Ure2p 1–79 (10 µM) assembly at 15°C followed by CD. The spectra recorded at time 0, 10, 20, 30, 40 and 60 min, purple – red, respectively, are shown. The CD spectra of soluble (red dashed line) and fibrillar (blue dashed line) α-synuclein (720 µM) are also shown. An isodichroic point is observed at 208 nm for Ure2p, 207 nm for α-synuclein. **B**, β-sheets formation upon the assembly of Ure2p 1–79 was derived from comparison of the changes in the CD spectra of Ure2p 1–79 and α-synuclein (which structure in amyloid fibrils is known) during assembly at 220 (○) and 200 (•) nm for β-sheets and random coil structures, respectively. The same assembly kinetic monitored by ThT binding is shown in **C**.

The assembly of soluble Ure2p 1–79 into fibrils was followed by measurement of the circular dichroism. A spectrum of typical random coils is observed during the early stages of assembly i.e. less than 30 minutes after Tobacco Etch Virus protease (TEV) treatment (Figure 4A, red hair-line), with a minimum of ellipticity at 198 nm. Later measurements indicate a decrease in ellipticity at 198 nm and an increase in ellipticity at 220 nm (corresponding to a β-sheet signal) over 150 minutes. An isobestic point is observed at 207 nm, indicative of a system with two temperature-independent components in equilibrium [24]. The time courses of ellipticity variation at 198 and 220 nm (figure 4B) correlate with ThT binding (figure 4C).

To assess the secondary structure changes that accompany Ure2p 1–79 assembly into fibrils, the recorded spectra were deconvoluted using the software CDSSTR [25] and soluble protein reference dataset spectra. The secondary structure content of soluble Ure2p 1–79 spectra at the early stages of assembly consist of 19% β-sheet, 22% α-helix and 60% disordered while that of fibrillar Ure2p 1–79 is 17% β-sheet, 53% α-helix, 30% disordered. The high α-helical contribution comes presumably from a helical fibrillar scaffold that impacts the CD spectrum, and the use of inappropriate CD reference set of β-rich proteins spectra for deconvolution [26]. To overcome this bias, we compared soluble and fibrillar Ure2p 1–79 spectra to that of soluble and fibrillar α-synuclein. The secondary structure content of the amyloid form of α-synuclein was determined recently with accuracy from solid-state NMR measurements [27] it consists of 27% of β-sheet and 73% other. Comparison of the molar ellipticity signals recorded upon the assembly of Ure2p 1–79 and full-length α-synuclein allowed us estimate the β-sheet content of fibrillar Ure2p 1–79 to 39%, consistent with FTIR measurements.

### The fibrillar forms of Ure2p and its N-terminal fragments and their respective seeding capacities

The prion concept is based on the capacity of minute amounts of aggregated prion molecules to convert the soluble form into high molecular weight aggregates [28]. Fibrils made *in vitro* of Ure2p prion domain induce [*URE3*] trait appearance when introduced in the cytoplasm of *S. cerevisiae* [22]. This is either the consequence of full-length Ure2p conversion into fibrils *via* a seeding process or the *de novo* induction of [*URE3*] by depletion of specific molecular chaperones by the fibrillar form of the prion domain of Ure2p and perturbed molecular chaperone homeostasis.

**Figure 5:**
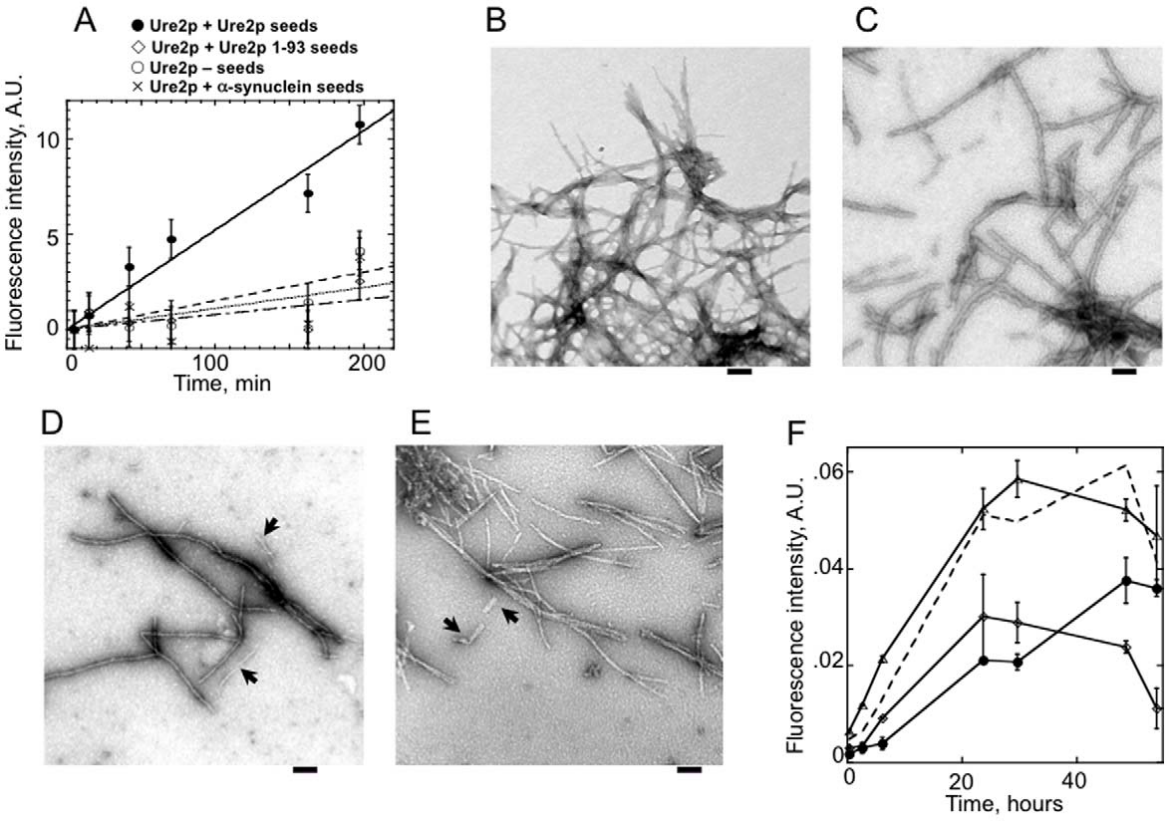
Cross-seeding capacities of fibrillar full-length Ure2p and Ure2p 1–93. **A**, Elongation of preformed full-length Ure2p fibrils, 2.5 µM (•) and Ure2p 1–93, 2.5 µM (⋄) in the presence of soluble full-length Ure2p, 50 µM monitored by ThT binding. The elongation reactions were performed at 15°C. The assembly of soluble full-length Ure2p in the absence of added seeds (○) and the presence of preformed α-synuclein fibrils, 2.5 µM (×) are shown as controls. Electron micrographs of assembly reaction products of **B**, Ure2p 1–93 alone, **C**, full-length Ure2p alone, **D**, full-length Ure2p in the presence of preformed fibrillar Ure2p 1–93 and **E**, Ure2p 1–93 in the presence of preformed fibrillar full-length Ure2p. **F**, assembly of soluble full-length Ure2p, 50 µM (•), Ure2p 1–93, 50 µM (⋄) and full-length Ure2p and Ure2p 1–93, 50 µM each (▵) monitored by ThT binding. The dashed line corresponds to the sum of the ThioT fluorescence recorded upon assembly of Ure2p 1–93 and soluble full-length Ure2p. The error bars in A and F indicate the standard deviation in three independent measurements.

To distinguish between the two possibilities, we assayed *in vitro* the ability of the fibrillar form of Ure2p 1–79 to seed the assembly of full-length Ure2p. The assembly kinetics, followed by ThT binding, were compared to that where full-length Ure2p fibrils and fibrils sharing no primary structure similarity with the prion domain of Ure2p e.g. α-synuclein fibrils are used to seed the assembly of soluble Ure2p. The initial elongation rates presented in figure 5A show that fibrillar full-length Ure2p seed most efficiently soluble full-length Ure2p assembly. Ure2p 1–93 fibrils in a manner similar to a control reaction containing fibrillar α-synuclein seeds do not induce the assembly of soluble Ure2p. We conclude from this observation that the limited induction of Ure2p assembly we observe upon addition to fibrillar Ure2p 1–93 or α-synuclein is not due to a nucleating activity *per se* but rather due to an unspecific process such as those observed upon addition of PEG in protein solutions upon protein crystallization assays [29]. The assembly of soluble Ure2p into protein fibrils was significantly accelerated, although to a lesser extent than in the presence of identical concentrations of fibrillar full-length Ure2p, upon addition of higher Ure2p 1–93 and α-synuclein seed concentrations (Figure S3). Nanoparticles and charged polysaccharides have been proposed to adsorb proteins, thus either affecting their conformational state or their local concentration, and as a consequence their assembly properties [30]–[32]. Ure2p assembly induction by elevated concentration of preformed Ure2p 1–93 fibrils which is of a similar extent to that of the unrelated α-synuclein fibrils is consistent with this view and suggests it is not due to the nucleation of Ure2p by preformed Ure2p1–93 fibrils.

To further determine whether the induction of full-length Ure2p assembly observed upon dilution of preformed Ure2p 1–93 and α-synuclein fibrils is due to a seeding activity, we examined the polymers in the electron microscope. Given that Ure2p 1–93 fibrils are 5 nm wide (Figure 5B) while full-length Ure2p fibrils are 25 nm wide (Figure 5C) one would expect to see if Ure2p 1–93 fibrils seed full-length Ure2p assembly thick fibrils with thin ends. Similarly, thin fibrils with thick ends should be observed if fibrillar full-length Ure2p was capable of seeding soluble Ure2p 1–93 assembly. None of the polymers we observed had such appearance in the electron microscope. In contrast, thin and thick fibrils were found to be lying next to each other (Figure 5, D and E). This suggests that the full-length Ure2p assembly induction observed upon addition of preformed Ure2p 1–93 and α-synuclein fibrils is unspecific, due to a crowding effect. To further determine whether Ure2p 1–93 co-assemble with full-length Ure2p, soluble Ure2p 1–93 were mixed with soluble full-length Ure2p and the assembly reaction was monitored by ThT binding. The assembly kinetics of full-length Ure2p alone or in the presence of Ure2p 1–93 as well as that of Ure2p 1–93 alone are presented in figure 5F. The data show in an unquestionable manner that the assembly kinetics are additive. Given that the assembly of Ure2p 1–93 is faster than that of full-length Ure2p two phases accounting for the independent assembly of each polypeptide are observed. This conclusion is further confirmed upon observation in the electron microscope of the fibrils generated *in vitro*. While thin fibrils made of Ure2p 1–93 are observed in the early stages of assembly, a mixed population of thin and thick fibrils is observed during the late stages of assembly (Figure S4). This further confirms that Ure2p 1–93 neither co-assemble with nor seed full-length Ure2p.

## Discussion

In the present study we show that the prion domain of Ure2p in whole or in parts assemble into fibrils of amyloid nature. In contrast, the full-length prion assembles into helical fibrils. Thus, the intrinsic assembly propensity of Ure2p prion domain into fibrils of amyloid nature appears to be abolished by its covalent association with the functional domain of the protein that leads to the generation of fibrils with high α-helical content. The counteracting activity of Ure2p C-terminal domain strongly suggests the existence of a crosstalk between the two protein moieties. The latter could theoretically lead to the acquisition of a defined structure by the N-terminal domain. Comparison of the CD difference spectra of full-length Ure2p and Ure2pC-terminal domain [23] and that of the GST-prion domain fusion and GST reveal however that Ure2p prion domain is poorly structured whether in its natural context or fused to the C-terminal end of GST *i.e.* establishing interaction with the rest of the protein or not, respectively.

The finding that Ure2p 42–79 has a higher propensity to assemble into amyloid fibrils than Ure2p 1–42 suggests that the latter polypeptide stretch modulates aggregation in its natural context in a negative manner. This agrees with the finding that amino-acid residues centred around residue 6 interact with those centred around residue 137 in assembly competent Ure2p [19]. The vast majority of natively unfolded polypeptides have been shown to form fibrils of amyloid nature. The finding that free Ure2p 42–79 readily forms fibrils of amyloid nature strongly suggests this peptide is natively unfolded. In contrast, the complex FTIR spectra recorded for free Ure2p 1–42 assemblies suggest that the peptide retain elements of secondary structure. Taken together our observations suggest that the N-terminal half of Ure2p prion domain not only interact with the C-terminal moiety of the prion protein but also possess elements of secondary structure.

It is worth noting that GST-Ure2p 1-42 has a higher assembly propensity than free Ure2p 1-42. It is possible that the ensemble of conformational states adopted by free Ure2p 1–42 is wider than that the polypeptide can explore when it is physically attached to the C-terminal end of GST. Thus, one reasonable explanation for this observation is that Ure2p 1–42 in GST-Ure2p 1–42 populates an assembly competent state faster than free Ure2p 1–42 because of its limited freedom and assemble more readily, as measured using thioflavin T binding.

Prions propagate by incorporation of soluble prion proteins into high molecular weight, fibrillar, oligomers. As the formation of stable nuclei following the conformational change that yield assembly competent prions is rate limiting, preformed prion aggregates seed very efficiently the assembly of soluble prions into fibrillar structures. The fibrillar form of full-length Ure2p generated under physiological experimental conditions seeds very efficiently the assembly of intact Ure2p. In contrast, fibrils made of the prion domain of the protein lack the ability to incorporate full-length Ure2p. This indicates that Ure2p N-terminal domain within its natural context does not populate a conformational state that incorporate within the amyloids that are generated when the prion domain is free in solution and out of its physiological context. This further support the model we proposed several years ago where the assembly of the prion Ure2p is driven by the establishment of intermolecular interactions between the N- and C-terminal domains of two consecutive Ure2p dimers and where the functional C-terminal domain of the protein is tightly involved in the fibrillar scaffold. Indeed, if the alternative model where assembly is driven by the stacking of Ure2p N-termini into a systematically H-bonded β-sheet core running along the fibrils to which the C-terminal domain is attached through a flexible region [33] was correct, amyloids made of free Ure2p N-terminal domain would seed the assembly of full-length Ure2p.

Fibrils made *in vitro* of Ure2p N-terminal domain were shown recently to induce, although to a lesser extent than those made of full-length Ure2p, [*URE3*] occurrence when reintroduced in yeast cells [22]. The results we report in this study suggest that [*URE3*] occurrence might be the consequence of the conversion of soluble cytosolic Ure2p into an insoluble form upon introduction of fibrils made of full-length Ure2p as fibrillar Ure2p seeds very efficiently the assembly of full-length Ure2p. Given that the amyloid fibrils made of the N-terminal domain of the protein i-exhibit no full-length Ure2p seeding capacity, ii- most likely expose to the solvent surface areas that differ from those exposed by full-length Ure2p fibrils, the process by which they induce [*URE3*] must differ from that of full-length Ure2p fibrils. The occurrence of [*URE3*] has been reported to be highly dependent on the expression levels of a number of molecular chaperones [34]–[37]. In addition, we have shown that the assembly of Ure2p is finely tuned by molecular chaperones *in vitro* [38]. Thus, it is possible that the induction of the prion trait upon reintroduction of fibrils made of Ure2p N-terminal domain is the consequence of their interaction interaction with molecular chaperones leading to *de novo* occurrence of [*URE3*]. The latter considerations and the finding that the [*URE3*] induction efficiency by full-length Ure2p fibrils is higher than that of fibrils made of the N-terminal domain of the protein [22] might thus reflect two independent pathways leading to a similar end point observable.

Further characterization of the affinity of molecular chaperone for fibrillar full-length Ure2p and Ure2p N-terminal domain using binding measurements and proteomic analysis should allow establishing whether the differences in the efficiency of [*URE3*] induction recorded for the two kinds of fibrils is due to two independent pathways leading to prion trait occurrence. This will undoubtedly contribute to a better understanding of the molecular events leading to [*URE3*] occurrence and propagation.

## Materials and Methods

### Construction of expression vectors and expression of Ure2p and related fusion proteins

Full length, wild type Ure2p (Ure2p) was expressed in *E.coli* BL21 DE3 codon plus strain (Stratagene™) and purified as described previously [13]. The N-terminal fragments of the prion domain spanning residues 1–42, 43–79, 1–79 and 1–93 were produced fused to the C-terminal end of GST with a TEV protease [39] cleavage site engineered between the GST and the N-terminal fragments of Ure2p (Figure 1A). PCR amplified fragments using specific primer (table S1) were subcloned into pETM-30 vector (EMBL, Heidelberg). The PCR products were digested by NdeI and NcoI, and ligated in pETM30 vector digested with the same enzymes. All construct were sequenced. The fusion geometry allows the production of soluble undegraded N-terminal fragments of the Ure2p prion domain.

Ure2p and the different fusion proteins were expressed in *E.coli* stain BL21 DE3 codon plus by addition of 0.5 mM isopropyl β-D-1-thiogalactopyranoside (IPTG). The bacterial pellets were resuspended in lysis buffer (Tris pH 7.5 10 mM, EDTA 1 mM, 0.1 mM phenylmethylsulfonyl fluoride (PMSF)). The cells were disrupted by sonication at 4°C. The lysate was clarified by centrifugation (25 minutes at 4°C, 20000 g) and loaded on the appropriate column for protein purification.

### Protein purification

Full-length Ure2p and α-synuclein were purified as previously described [13], [23], [40]. The GST-N-terminal fragments of Ure2p prion domain were purified using a 10 ml Glutathione sepharose 4 fast flow column (GE healthcare). The column was washed with 100 ml of buffer A (Tris pH 7.5 10 mM, KCL 200 mM, 1 mM beta-mercaptoethanol (βME)) after the samples were loaded and was eluted with 10 ml of buffer A supplemented with 10 mM Glutathione. Fraction containing the fusion proteins were immediately aliquoted, flash-frozen in liquid nitrogen, and stored at −80°C.

To generate free Ure2p 1–42, 42–79, 1–79 and 1–93, the fusion proteins were immobilized on a 10 ml Glutathione sepharose column. The column was filled with 6xHis-tagged TEV protease (0.3 mg/ml in Buffer A). The column was then washed with buffer A and the TEV protease was remove inline by a 5 ml His-trap chelating column (GE healthcare). Fraction containing pure free Ure2p 1–42, 42–79, 1–79 and 1–93 were immediately aliquoted, flash-frozen in liquid nitrogen, and stored at −80°C.

All purified Ure2p N-terminal fragments and fusion proteins were checked by MALDI-TOF mass spectrometry. Purified Ure2p 1–42, 42–79, 1–79 and 1–93 analysed on Tris-Tricine SDS PAGE exhibit an abnormal migration with higher apparent molecular weights and stain pink upon staining with Coomassie blue R250 (not shown).

### Assembly of full-length Ure2p and Ure2p 1**–**42, 42**–**79, 1**–**79 and 1**–**93 into protein fibrils, seeding and cross-seeding experiments

The assembly of full-length Ure2p, Ure2p 1–42, 42–79, 1–79 and 1–93 was achieved by incubation of the proteins at 6°C without shaking in buffer A. The assembly reaction was monitored using ThT binding [41] using a Cary Eclipse fluorescence spectrophotometer (Varian Inc., Palo Alto, CA). The elongation rates of preformed fibrillar Ure2p 1–42, 42–79, 1–79 and 1–93 in the presence of soluble full-length Ure2p and of Ure2p fibrils in the presence of soluble Ure2p 1–42, 42–79, 1–79 and 1–93 were measured upon diluting the preformed fibrils in the presence of the soluble proteins at a molar ratio 5∶100. Fibrillar α-synuclein was added to control reaction to assess the unspecific effect of fibrils addition on Ure2p and its N-terminal fragments assembly reactions.

Assemblies were examined following negative staining with 1% Uranyl acetate on carbon-coated grids (200 mesh) in a Jeol 1400 transmission electron microscope. Images were recorded with a Gatan Orius CCD camera (Gatan, Pleasanton, CA).

### X-ray fiber diffraction measurements

The fibrillar samples were spun 13 000 rpm for 15 min at 4°C. The pellets were resuspended twice in distilled water. Pelleted fibrils were introduced into a 1 mm diameter glass capillary and dehydrated at room temperature for two days. X-ray fiber diffraction patterns were collected on a MAR345 image plate (MArresearch Gmbh, Germany) using a Rigaku RU200 rotating-anode generator source (beam size 100×100 µm). The acquisition parameters were set to 300 mm sample-to-detector distance, and 20-minute exposure. Images were processed by Mosflm [42] and FibreFix version 1.3.1 software (http:/www.fibre-diffraction.ac.uk).

### Fourier transform infrared spectrometry

Fibrillar samples (2 mg) were spun at 16000 g at 4°C for 10 min and resuspended in 100 µl D_2_O. Following a second centrifugation/resuspension step, infrared spectra were recorded on a JASCO 660 Plus FTIR spectrometer equipped with a Mercury Cadmium Telluride (MCT) detector using the attenuated total reflectance mode. The background consisted of D_2_O. A total of 256 interferograms were collected with a resolution of 1 cm^−1^.

### Circular dichroism

The CD of GST-Ure2p 1–42, GST-Ure2p 1–79 and GST-Ure2p 1–93 were recorded at 20°C using a JASCO J810 dichrograph using 1 mm pathlength quartz cuvettes (Hellma) containing 150 µl of the protein solutions. Synchrotron Radiation Circular Dichroism (SRCD) of free Ure2p 1–79 was recorded at 15°C on UV I beamline at ISA (Aarhus university synchrotron radiation facility, Aarhus, DK). Samples were loaded into a 500 µm pathlength suprasil round cell (HELLMA). Acquisitions at 1 nm steps, between 280 to 175 nm were performed in triplets. Averaged sample spectra subtracted from their corresponding buffer Baseline. Spectral magnitude was veryfied using a solution of (+)-camphour-10-sulphonic acid (CSA).

All spectra were normalized to the mean residue weight ellipticity (θMRW) [deg cm2/dmole] using the equation θ(λ)MRW  =  θ(λ)mdeg/10·c·n·d where θ(λ)mdeg is the recorded spectra in millidegrees, n is the number of amino acid residues, c is the sample concentration in moles per liter and d is the path length of the cuvette in centimeters.

Accurate protein concentration measurements were done by quantitative amino acid analysis (QAA) performed at the Institut Pasteur (Unit Chemistry of Biomolecules).

## Supporting Information

Figure S1 (3.00 MB TIF)

Figure S2 (3.00 MB TIF)

Figure S3 (3.00 MB TIF)

Figure S4 (3.00 MB TIF)

Table S1 (0.02 MB DOC)

## References

[1] Prusiner SB (1982). Novel proteinaceous infectious particles cause scrapie.. Science.

[2] Cox BS (1965). PSI, a cytoplasmic suppressor of super-suppressor in yeast.. Heredity.

[3] Aigle M, Lacroute F (1975). Genetic aspects of [URE3] a non-Mendelian cytoplasmically inherited mutation in yeast.. Mol Gen Genet.

[4] Derkatch IL, Bradley ME, Zhou P, Chernoff YO, Liebman SW (1997). Genetic and environmental factors affecting the *de novo* appearance of the [PSI+] prion in Saccharomyces cerevisiae.. Genetics.

[5] Tuite MF, Cox BS (2003). Propagation of yeast prions.. Nat Rev Mol CellBiol.

[6] Bousset L, Savistchenko J, Melki R (2008). Assembly of the asparagine- and glutamine-rich yeast prions into protein fibrils.. Curr Alzh Res.

[7] Redeker V, Halgand F, Le Caer JP, Bousset L, Laprevote O (2007). Hydrogen/deuterium exchange mass spectrometric analysis of conformational changes accompanying the assembly of the yeast prion Ure2p into protein fibrils.. J Mol Biol.

[8] Toyama BH, Kelly MJ, Gross JD, Weissman JS (2007). The structural basis of yeast prion strain variants.. Nature.

[9] Sondheimer N, Lindquist S (2000). Rnq1: an epigenetic modifier of protein function in yeast.. Mol Cell.

[10] Maddelein ML, Wickner RB (1999). Two prion-inducing regions of Ure2p are nonoverlapping.. Mol Cell Biol.

[11] Kurahashi H, Ishiwata M, Shibata S, Nakamura Y (2008). A regulatory role of the Rnq1 nonprion domain for prion propagation and polyglutamine aggregates.. Mol Cell Biol.

[12] Liu JJ, Sondheimer N, Lindquist SL (2002). Changes in the middle region of Sup35 profoundly alter the nature of epigenetic inheritance for the yeast prion [*PSI*+].. Proc Natl Acad Sci USA.

[13] Thual C, Komar AA, Bousset L, Fernandez-Bellot E, Cullin C (1999). Structural characterization of Saccharomyces cerevisiae prion-like protein Ure2.. J Biol Chem.

[14] Masison DC, Wickner RB (1995). Prion-inducing domain of yeast Ure2p and protease resistance of Ure2p in prion-containing cells.. Science.

[15] Ranson N, Stromer T, Bousset L, Melki R, Serpell LC (2006). Insights into the architecture of the Ure2p yeast protein assemblies from helical twisted fibrils.. Protein Sci.

[16] Bousset L, Thomson NH, Radford SE, Melki R (2002). The yeast prion Ure2p retains its native alpha-helical conformation upon assembly into protein fibrils *in vitro*.. EMBO J.

[17] Bousset L, Briki F, Doucet J, Melki R (2003). The native-like conformation of Ure2p in fibrils assembled under physiologically relevant conditions switches to an amyloid-like conformation upon heat-treatment of the fibrils.. J Struct Biol.

[18] Bousset L, Redeker V, Decottignies P, Dubois S, Le Marechal P (2004). Structural characterization of the fibrillar form of the yeast Saccharomyces cerevisiae prion Ure2p.. Biochemistry.

[19] Fay N, Redeker V, Savistchenko J, Dubois S, Bousset, L (2005). Structure of the prion Ure2p in protein fibrils assembled *in vitro*.. J Biol Chem.

[20] Loquet A, Bousset L, Gardiennet C, Sourigues Y, Wasmer C (2009). Prion fibrils of Ure2p assembled under physiological conditions contain highly ordered, natively folded modules.. J Mol Biol.

[21] Baxa U, Wickner RB, Steven AC, Anderson DE, Marekov LN, et el (2007). Characterization of beta-sheet structure in Ure2p1–89 yeast prion fibrils by solid-state nuclear magnetic resonance.. Biochemistry.

[22] Brachmann A, Baxa U, Wickner RB (2005). Prion generation in vitro: amyloid of Ure2p is infectious.. EMBO J.

[23] Thual C, Bousset L, Komar AA, Walter S, Buchner J (2001). Stability, Folding, Dimerization, and Assembly Properties of the Yeast Prion Ure2p.. Biochemistry.

[24] Sandstrom J, Berova N, Nakanishi K, Woody RW (2000). In Circular dichroism principles and applications, second edition..

[25] Johnson WC (1999). Analyzing protein circular dichroism spectra for accurate secondary structures.. Proteins.

[26] Evans P, Bateman OA, Slingsby C, Wallace BA (2007). A reference dataset for circular dichroism spectroscopy tailored for the betagamma-crystallin lens proteins.. Exp Eye Res.

[27] Heise H, Celej MS, Becker S, Riedel D, Pelah A (2008). Solid-state NMR reveals structural differences between fibrils of wild-type and disease-related A53T mutant alpha-synuclein.. J Mol Biol.

[28] Castilla J, Saa P, Hetz C, Soto C (2005). In vitro generation of infectious scrapie prions.. Cell.

[29] Geigé R, Ducruix A (1992). In Ducruix A and Giegé R (eds.), Crystallization of Nucleic Acids and Proteins.. A Practical Approach..

[30] Linse S, Cabaleiro-Lago C, Xue WF, Lynch I, Lindman S (2007). Nucleation of protein fibrillation by nanoparticles.. Proc Natl Acad Sci USA.

[31] Colvin VL, Kulinowski KM (2007). Nanoparticles as catalysts for protein fibrillation.. Proc Natl Acad Sci USA.

[32] Motamedi-Shad N, Monsellier E, Torrassa S, Relini A, Chiti F (2009). J Biol Chem.

[33] Kajava AV, Baxa U, Wickner RB, Steven AC (2004). A model for Ure2p prion filaments and other amyloids: the parallel superpleated beta-structure.. Proc Natl Acad Sci USA.

[34] Moriyama H, Edskes, HK, Wickner RB (2000). [URE3] prion propagation in Saccharomyces cerevisiae: requirement for chaperone Hsp104 and curing by overexpressed chaperone.. Ydj1p Mol Cell Biol.

[35] Schwimmer C, Masison DC (2002). Antagonistic interactions between yeast [PSI(+)] and [URE3] prions and curing of [URE3] by Hsp70 protein chaperone Ssa1p but not by Ssa2p.. Mol Cell Biol.

[36] Roberts BT, Moriyama H, Wickner RB (2004). [URE3] prion propagation is abolished by a mutation of the primary cytosolic Hsp70 of budding yeast.. Yeast.

[37] Loovers HM, Guinan E, Jones GW (2007). Importance of the Hsp70 ATPase domain in yeast prion propagation.. Genetics.

[38] Savistchenko J, Krzewska J, Fay N, Melki R (2008). Molecular chaperones and the assembly of the prion Ure2p in vitro.. J Biol Chem.

[39] Kapust RB, Waugh DS (2000). Controlled intracellular processing of fusion proteins by TEV protease.. Prot Expr Purif.

[40] Ghee M, Melki R, Michot N, Mallet J (2005). PA700, the regulatory complex of the 26S proteasome, interferes with a-synuclein assembly.. FEBS J.

[41] McParland VJ, Kad NM, Kalverda AP, Brown A, Kirwin-Jones P (2000). Partially unfolded states of beta(2)-microglobulin and amyloid formation in vitro.. Biochemistry.

[42] Leslie AGW (1992).

