# Supplementary figures and images for: Structure and Assembly Properties of the N-Terminal Domain of the Prion Ure2p in Isolation and in Its Natural Context
Source: PLoS One. 2010 Mar 22;5(3):e9760. doi: 10.1371/journal.pone.0009760 (PMC2842292; doi:10.1371/journal.pone.0009760)

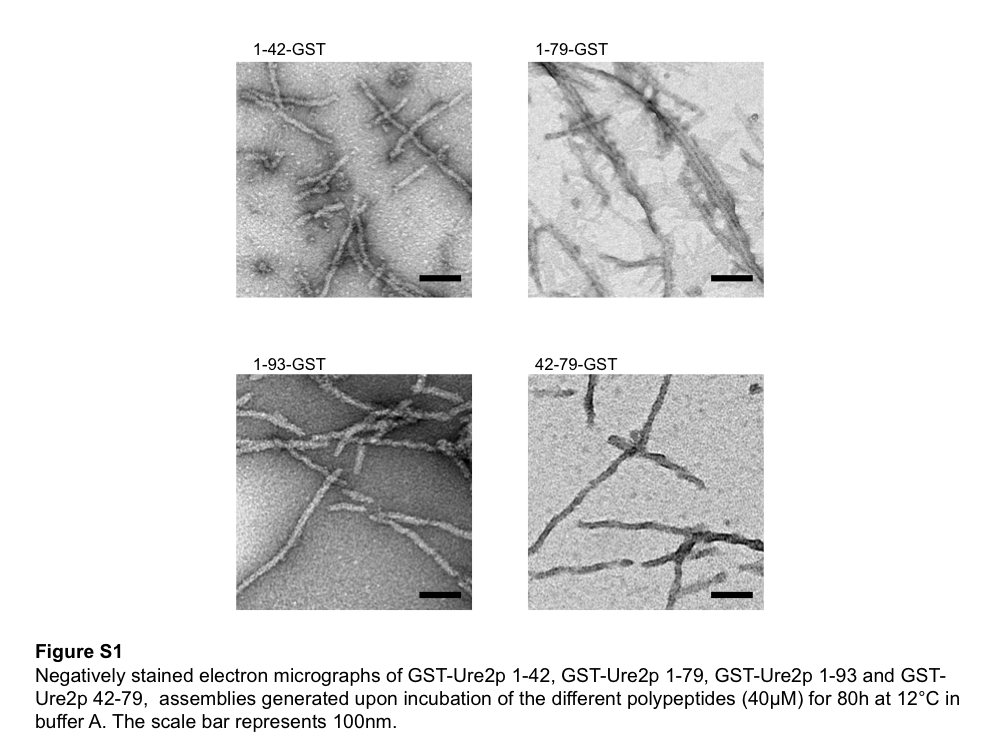

Supplement: Figure S1 — (3.00 MB TIF) [file pone.0009760.s001.tif]

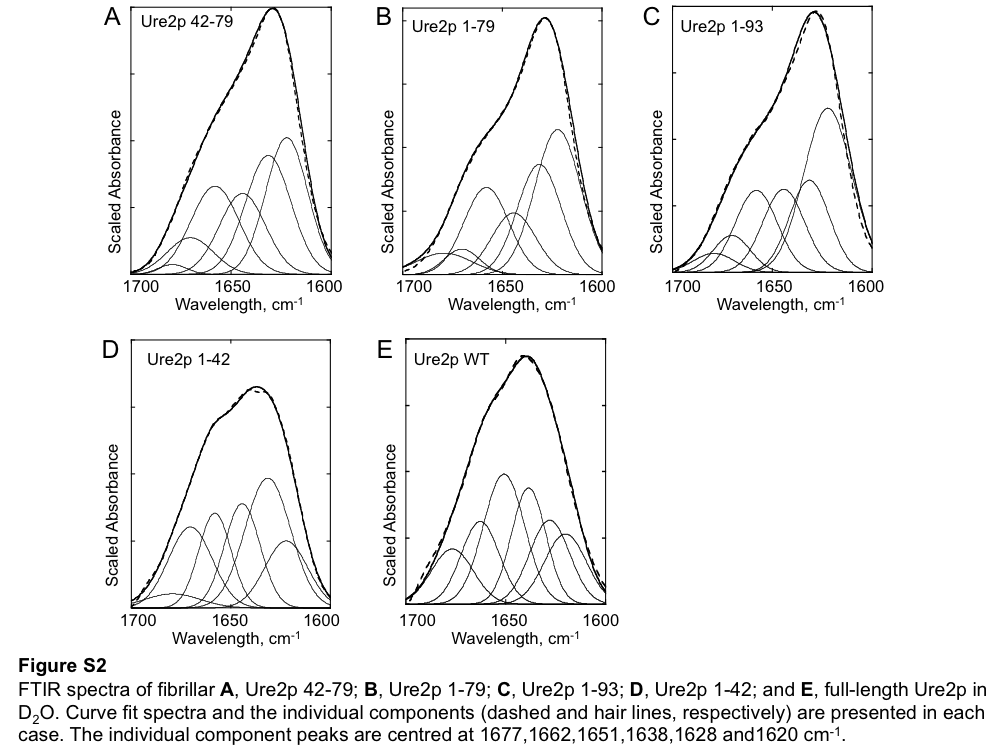

Supplement: Figure S2 — (3.00 MB TIF) [file pone.0009760.s002.tif]

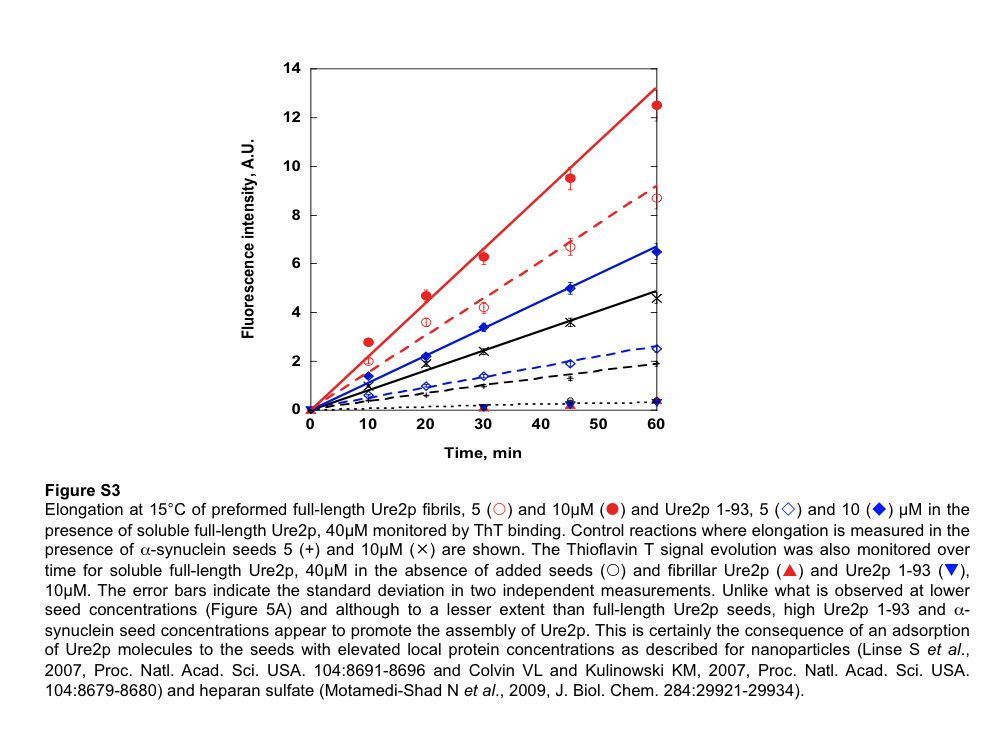

Supplement: Figure S3 — (3.00 MB TIF) [file pone.0009760.s003.tif]

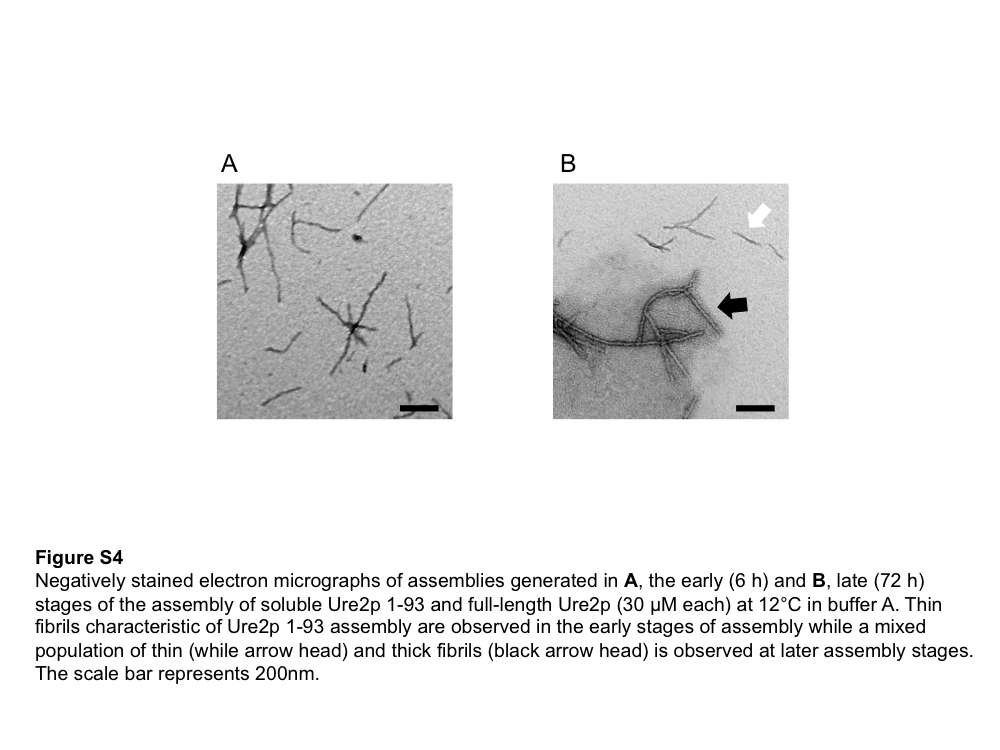

Supplement: Figure S4 — (3.00 MB TIF) [file pone.0009760.s004.tif]
