# Supplementary material for: Structure and Assembly Properties of the N-Terminal Domain of the Prion Ure2p in Isolation and in Its Natural Context
Source: PLoS One. 2010 Mar 22;5(3):e9760. doi: 10.1371/journal.pone.0009760 (PMC2842292; doi:10.1371/journal.pone.0009760)
Supplement: Table S1 — (0.02 MB DOC) [file pone.0009760.s005.doc]

**Supplementary INFORMATION**

**Supplementary Table 1.** Oligonucleotide sequences used to generate the different polypeptides from Ure2p N-terminal domain.

Forward primer for the generation of Ure2p 1-42, 1-79 and 1-93 5’- GGCCTGCCATGGTGAATAACAACGGCAACCAAGTGTCGAATCTCTCC-3’

Ure2p 1-42 reverse primer 5’- GGCCTGCCATGGTGAATAACAACGGCAACCAAGTGTCGAATCTCTCC-3’

Ure2p 1-79 reverse primer 5’- CGCGGATCCTCAATTCTTGATATTATTCTCGTTATCATTATTTTGGCTACC-3’

Ure2p 1-93 reverse primer 5’- CGCGGATCCTCAATCCGAAAATGCCTGTTGTTGTTGTCGATGTTG-3’

Ure2p 42-79 forward primer 5’-

ggcctgCCATGGGTGTAAATAATAATAATAATAACAATAGCAGTAGTAATAAC-3’
